# Genome-wide RNA-sequencing dataset reveals the prognostic value and potential molecular mechanisms of lncRNA in non-homologous end joining pathway 1 in early stage Pancreatic Ductal Adenocarcinoma

**DOI:** 10.7150/jca.39888

**Published:** 2020-07-20

**Authors:** Li-Ming Shang, Xi-Wen Liao, Guang-Zhi Zhu, Ke-Tuan Huang, Chuang-Ye Han, Cheng-Kun Yang, Xiang-Kun Wang, Xin Zhou, Hao Su, Xin-Ping Ye, Tao Peng

**Affiliations:** Department of Hepatobiliary Surgery, The First Affiliated Hospital of Guangxi Medical University, Nanning, 530021, Guangxi Zhuang Autonomous Region, People's Republic of China.

**Keywords:** lncRNA in non-homologous end joining pathway 1, pancreatic ductal adenocarcinoma, molecular mechanism, prognosis, The Cancer Genome Atlas

## Abstract

**Objective:** Our current study is to explore the prognostic value and molecular mechanisms underlying the role of lncRNA in non-homologous end joining pathway 1 (LINP1) in early stage pancreatic ductal adenocarcinoma (PDAC).

**Methods:** Genome-wide RNA-seq datasets of 112 early stage PDAC patients were got from The Cancer Genome Atlas and analyzed using multiple online tools.

**Results:** Overall survival in high LINP1 expression patients was shorter than those with low expression (high-LINP1 vs. low-LINP1=481 vs. 592 days, log-rank P=0.0432). The multivariate Cox proportional hazard regression model suggested that high-LINP1 patients had a markedly higher risk of death than low-LINP1 patients (adjusted P=0.004, hazard ratio=2.214, 95% confidence interval=1.283-3.820). Analysis of genome-wide co-expressed genes, screening of differentially expressed genes, and gene set enrichment analysis indicated that LINP1 may be involved in the regulation of cell proliferation-, cell adhesion- and cell cycle-related biological processes in PDAC. Six small-molecule compounds including STOCK1N-35874, fenofibrate, exisulind, NU-1025, vinburnine, and doxylamine were identified as potential LINP1-targeted drugs for the treatment of PDAC.

**Conclusions:** Our study indicated that LINP1 may serve as a prognostic biomarker of early stage PDAC. Analysis of genome-wide datasets led to the elucidation of the underlying mechanisms and identified six potential targeted drugs for the treatment of early PDAC.

## Introduction

The most common primary pancreatic cancer is a tumor that occurs in the exocrine part of the pancreas. It has a high degree of malignancy and a short disease course characterized by rapid development and deterioration. Treating this cancer is difficult because it is often only detected at a late stage, and the mortality rate is high 1. There are various treatment modalities for pancreatic cancer, including surgery, chemotherapy, radiotherapy and interventional therapy. However, the efficacy of single-modality treatment is limited because pancreatic cancer has a high degree of malignancy 1, 2. Most patients with pancreatic cancer are difficult to diagnose early; therefore, the resection rate is low, and the five years survival rate of patients undergoing tumor resection is <25% 3. Most of pancreatic cancer is pancreatic ductal adenocarcinoma (PDAC). Tumor occurrence is the result of multiple genetic and epigenetic abnormalities. Rapid advances in high-throughput sequencing technology led to the identification of long non-coding RNAs (lncRNAs) as key players in the occurrence, development, and prognosis of cancers 4. The large amount of high-throughput sequencing data obtained by The Cancer Genome Atlas (TCGA) is helpful for the screening and identification of cancer related biomarkers and therapeutic targets 5, 6. Previous studies suggest that lncRNA in non-homologous end joining pathway 1 (LINP1) plays an oncogenic role in tumors and is significantly correlated with tumor progression and prognosis 7-9. However, the value of LINP1 for clinical application in pancreatic cancer and its underlying mechanisms remain unclear. Our current study is to explore the prognostic value and potential molecular mechanisms of LINP1 in early stage PDAC.

## Materials and methods

### Data acquisition

The pancreatic cancer RNA-sequencing (RNA-seq) dataset included in the present study was got from TCGA data portal (https://portal.gdc.cancer.gov) 5, and the relevant clinical parameters were obtained from the University of California, Santa Cruz Xena (UCSC Xena:http://xena.ucsc.edu/) browser. Criteria for patient inclusion and exclusion were as described in our previous studies 10-12. The present study included 112 PDAC patients with an early stage 10-12. The acquisition and use of data in our study were in conformity to TCGA guidelines. Data were derived from TCGA, therefore additional ethics committee approval were not applicable.

### Survival analysis of LINP1 in early stage PDAC

Survival analysis of LINP1 in early stage PDAC patients was used the multivariate Cox proportional hazard regression model and Kaplan-Meier curve. A time-dependent receiver operating characteristic (ROC) curve was used to assess the power of LINP1 in predicting the prognosis of early stage PDAC patients through the *survivalROC* package. A nomogram was constructed to assess the contribution of LINP1 to PDAC prognosis prediction. The prognostic value of LINP1 in combination with clinical parameters in PDAC was evaluated using joint effects survival analysis.

### Functional assessment of LINP1 in early stage PDAC

The molecular mechanisms underlying the role of LINP1 in PDAC were investigated by identifying differentially expressed genes (DEGs) between low- and high-LINP1 expression groups. LINP1 co-expressed genes in PDAC were identified for functional enrichment analysis, which was input into the Database for Annotation, Visualization and Integrated Discovery (DAVID, https://david.ncifcrf.gov/home.jsp) v6.8 13, 14 and Biological Networks Gene Ontology tool (BiNGO) 15. The interactions between these genes were examined using GeneMANIA (http://genemania.org/)^16^, ^17^ and the Search Tool for the Retrieval of Interacting Genes/Proteins (STRING, https://string-db.org/) 18-20. DEGs between different LINP1 expression groups were screened using the *edgeR* package 21. Genes meeting the criteria of |log2 fold change (log2FC)|>1, *P* value less than 0.05 and FDR less than 0.05 were considered as DEGs. LINP1 co-expressing genes were identified by Pearson's correlation coefficient (P<0.05).

DEGs between different LINP1 expression groups were applied to the Connectivity Map (CMap, https://portals.broadinstitute.org/cmap/) online tool to predict and screen potential LINP1 targeted drugs in PDAC 22, 23. The structures of the compounds are available from the PubChem (https://pubchem.ncbi.nlm.nih.gov/) 24. The molecular mechanisms involved in PDAC different LINP1expression groups were examined by the gene set enrichment analysis (GSEA) approach 25. The reference gene set used by GSEA was got from the Molecular Signatures Database v6.2 (c2.all.v6.2.symbols.gmt, and c5.all.v6.2.symbols.gmt) 26, 27. Gene sets with nominal P-value less than 0.05, |Normalized Enrichment Score (NES)| greater than 1 and false discovery rate (FDR) less than 0.25 in the GSEA report were considered reach statistical significance.

### Statistical analysis

Kaplan-Meier curve was assessed by the log-rank test. Hazard ratio (HR) and 95% confidence interval (CI) was used to assess univariate and multivariate Cox proportional hazards regression model. P value < 0.05 was considered reach statistical significance. All data statistics were assessed by SPSS version 22.0 and R 3.5.0.

## Results

### Survival analysis of LINP1 in early stage PDAC

**Figure 1:**
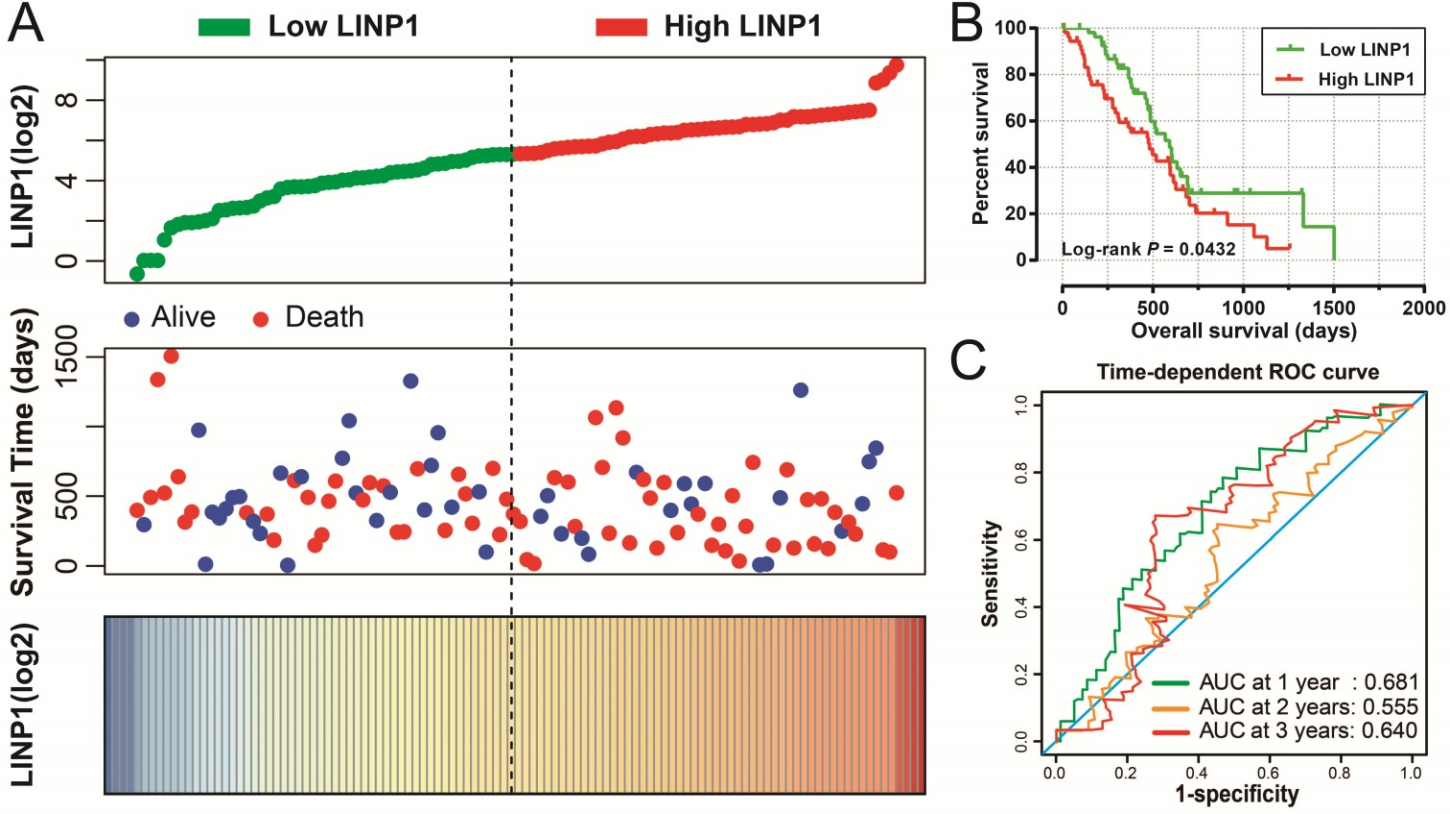
Survival analysis of LINP1 expression levels in early stage pancreatic adenocarcinoma (PDAC). (**A**) Relationship between LINP1 expression and OS in patients with PDAC. (**B**) Kaplan-Meier curve of the effect of LINP1 expression on OS in patients with PDAC. (**C**)Time dependent ROC curve of LINP1 for PDAC clinical outcome prediction.

**Figure 2:**
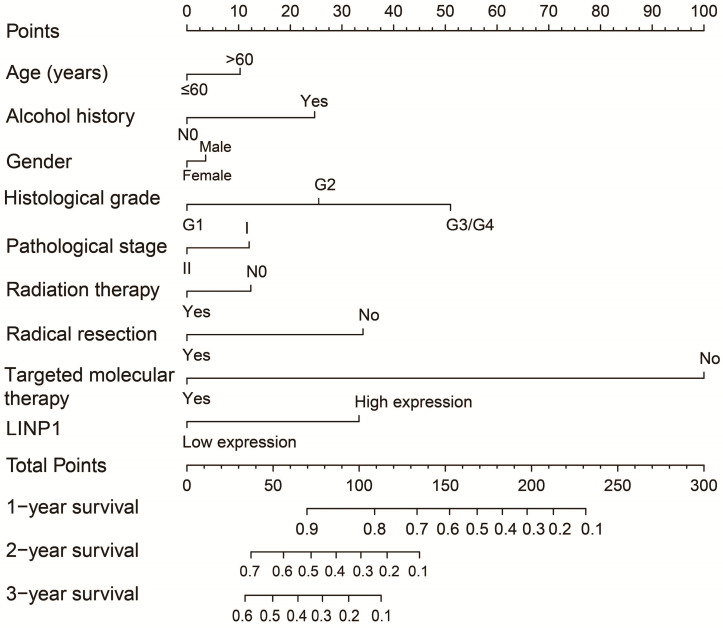
Nomogram of the combination of LINP1 with clinical parameters for predicting the prognosis of patients with early PDAC.

**Figure 3:**
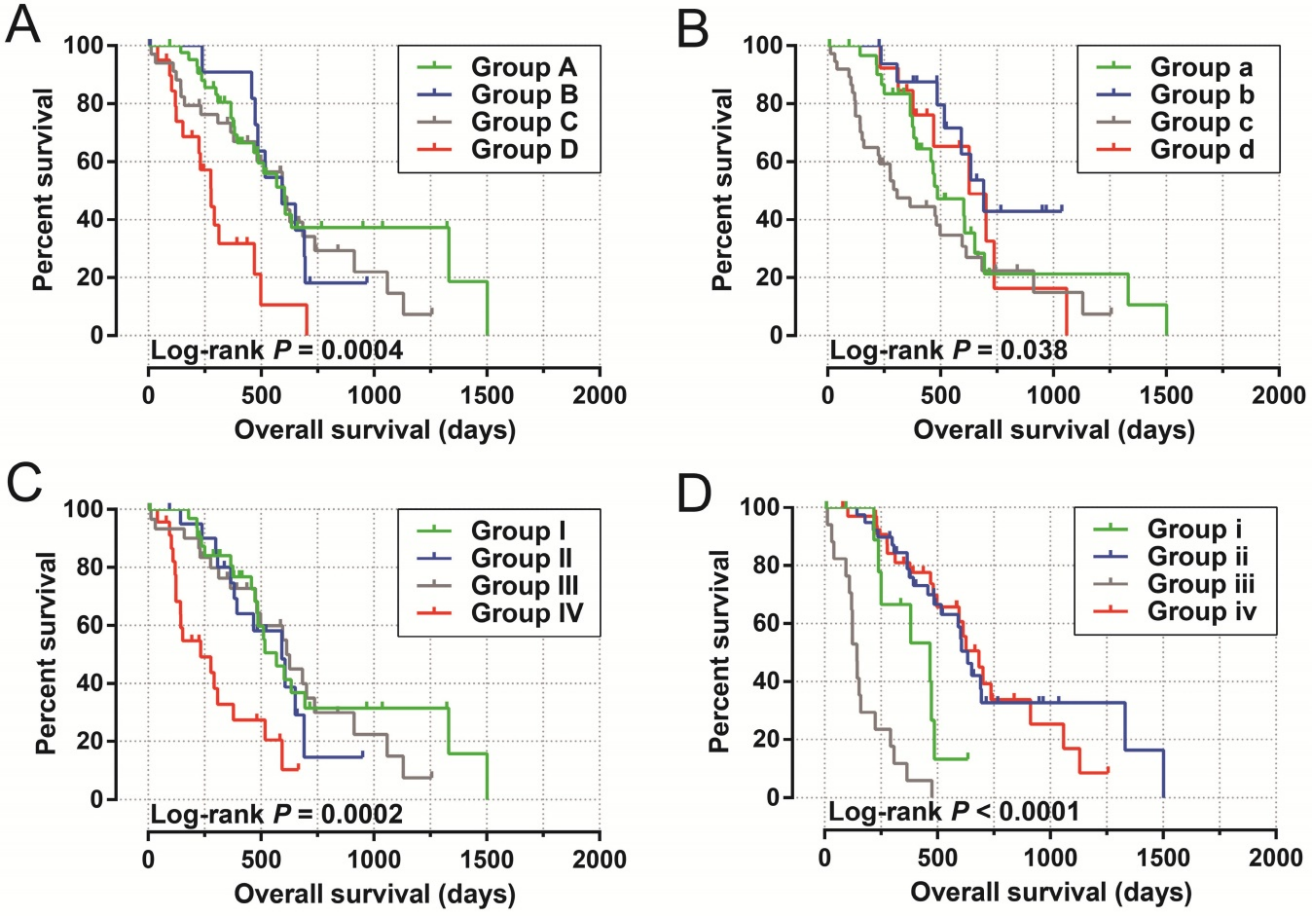
Joint effects survival analysis of LINP1 and clinical parameters in PDAC OS. (**A**) Histological grade; (**B**) radiation therapy; (**C**) radical resection; (**D**) targeted molecular therapy.

The clinical characteristics of 112 early stage PDAC patients are summarized in **Table S1**. Survival analysis suggested that high LINP1 expression patients had a poor prognosis compared with these with low expression (high-LINP1 vs. low-LINP1 = 481 vs. 592 days, log-rank *P* = 0.0432, **Figure 1A-B**). The multivariate Cox proportional hazard regression model suggested that high LINP1 expression patients had a notably increased risk of death compared with low LINP1 expression patients (adjusted* P* = 0.004; HR = 2.214; 95% CI = 1.283-3.820). Time-dependent ROC analysis indicated that LINP1 expression was a good predictor of 1 year survival in early stage PDAC with an area under the curve (AUC) of 0.681 (**Figure 1C**). A nomogram constructed using clinical parameters and LINP1 expression levels indicated that LINP1 contributed more than 30 points to the prognosis, which was higher than the score for clinical parameters such as radiation therapy and alcohol consumption history (**Figure 2**). Joint effects survival analysis of the value of LINP1 combined with clinical parameters for predicting the overall survival (OS) of patients with PDAC showed that LINP1 significantly improved the ability to predict prognosis (**Figure 3A-D, Table S2**).

### Genome-wide co-expression analysis of LINP1 in PDAC

**Figure 4:**
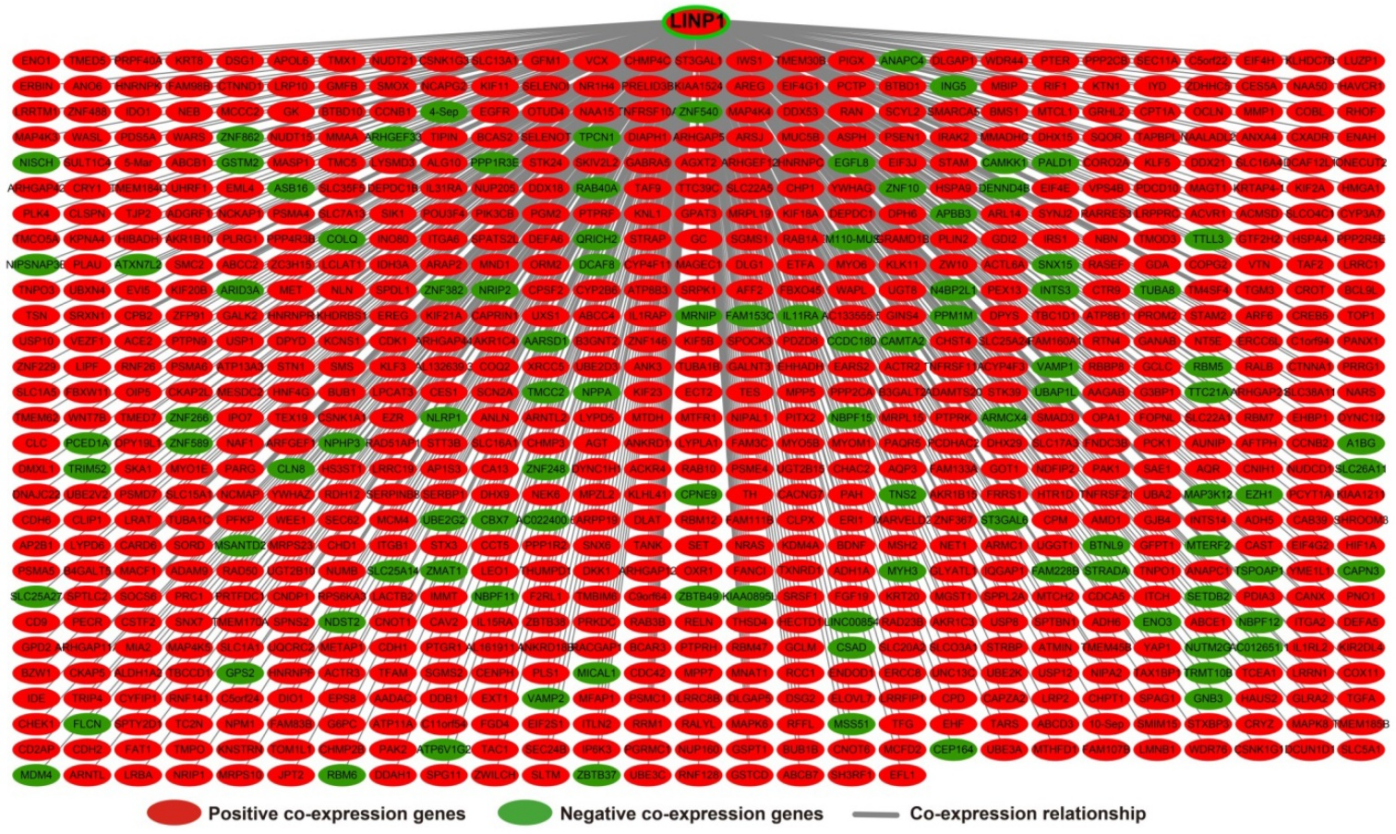
Regulatory network of LINP1 co-expressed genes in PDAC tumor tissues.

**Figure 5:**
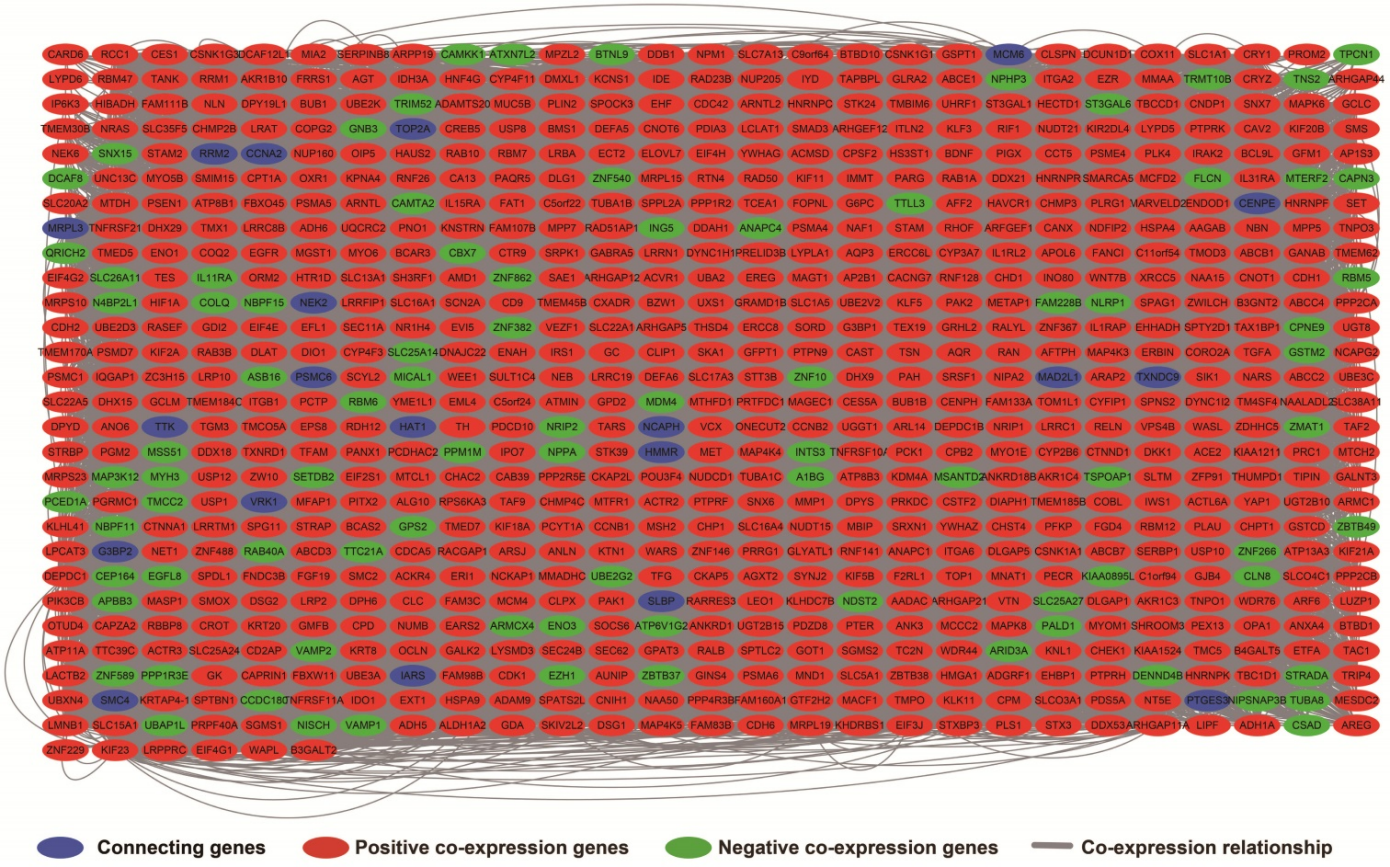
Gene-gene interaction regulatory network of LINP1 co-expressed genes.

**Figure 6:**
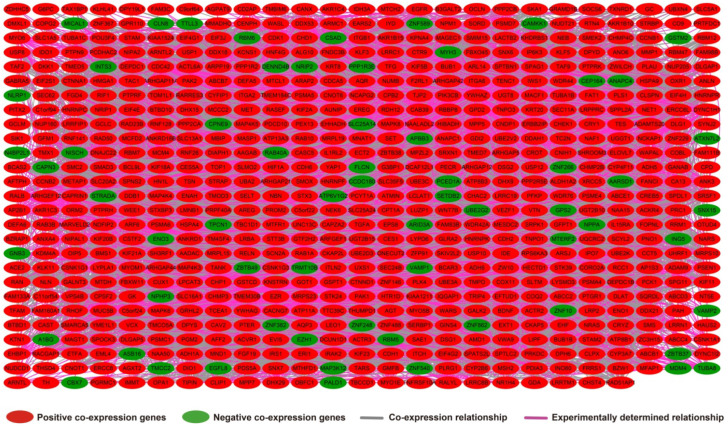
Protein-protein interaction regulatory network of LINP1 co-expressed genes.

The results of genome-wide co-expression analysis of LINP1 in PDAC tumor tissues are summarized in **Table S3**. A total of 774 genes were recognized as co-expressing protein coding genes of LINP1 in PDAC tumor tissues, of which 99 were negatively correlated and 675 were positively correlated (**Figure 4**). Gene Ontology (GO) term analysis revealed that LINP1 co-expressed genes may be play a role in biological processes, such as T cell receptor signaling pathway, G2/M transition of the mitotic cell cycle, cell-cell adherens junctions, cell division, cell-cell adhesion, the epidermal growth factor receptor (EGFR) signaling pathway, cell proliferation and the G2 DNA damage checkpoint (**Table S4**). The BINGO results also confirmed the above results, and indicated that LINP1 co-expressed genes may be play a role in the cell cycle, cell division, cell adhesion mediated by integrin, regulation of cell migration, MAPKKK cascade, and the EGFR signaling pathway (**Figure S1**). Kyoto Encyclopedia of Genes and Genomes (KEGG) analysis revealed that LINP1 co-expressed genes are play a role in the AMPK signaling pathway, adherens junction, proteoglycans in cancer, cell cycle, metabolism of xenobiotics by cytochrome P450, and the Hedgehog signaling pathway (**Table S5**). Construction of gene-gene interaction (GGI) (**Figure 5**) and protein-protein interaction (PPI) (**Figure 6**) regulatory networks using GeneMANIA and STRING online tools led to the identification of a complex network of regulatory relationships among LINP1 co-expressed genes.

### DEGs screening and functional enrichment analysis

**Figure 7:**
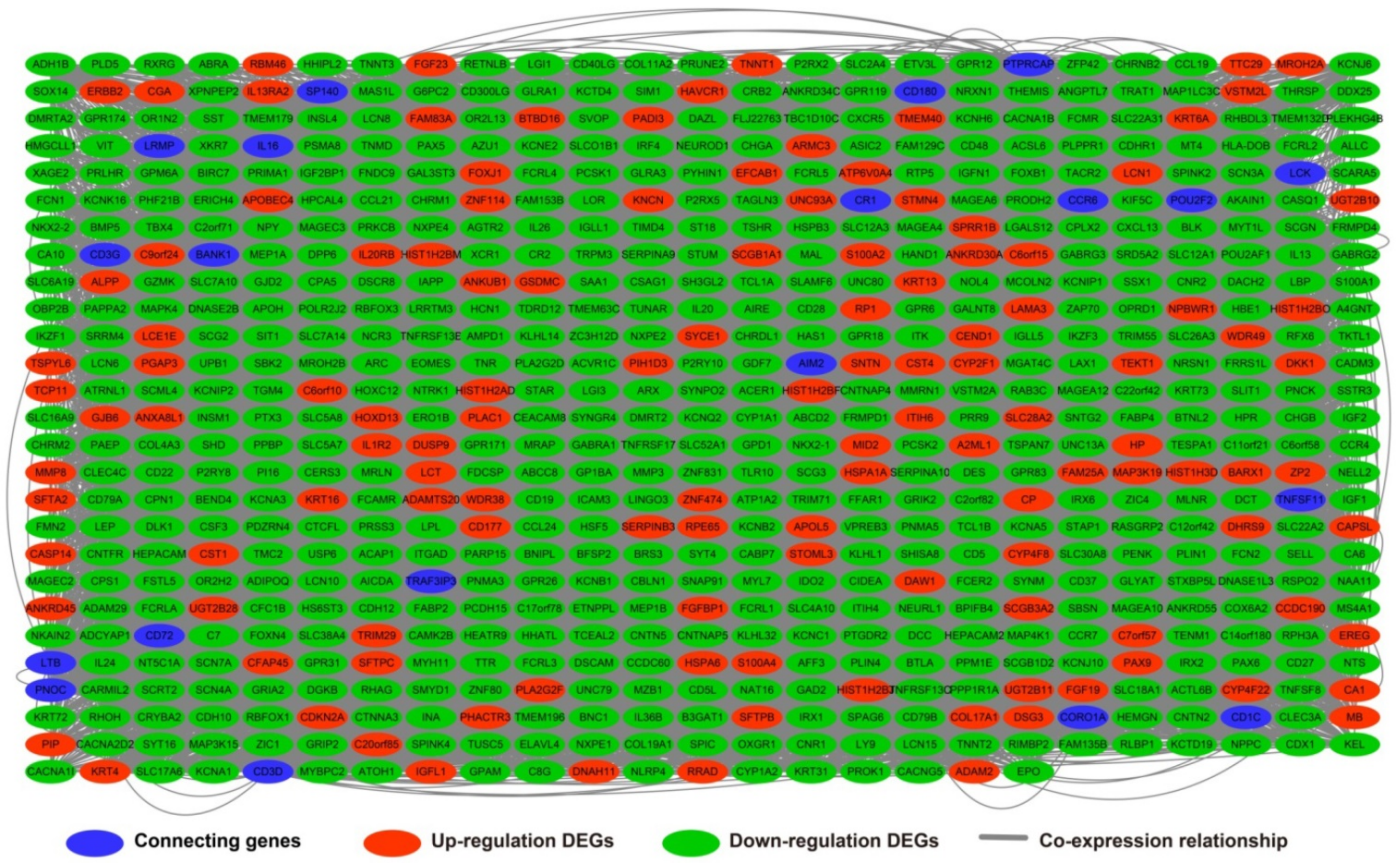
Gene-gene interaction regulatory network of differentially expressed genes (DEGs) between high- and low-LINP1 expression groups.

**Figure 8:**
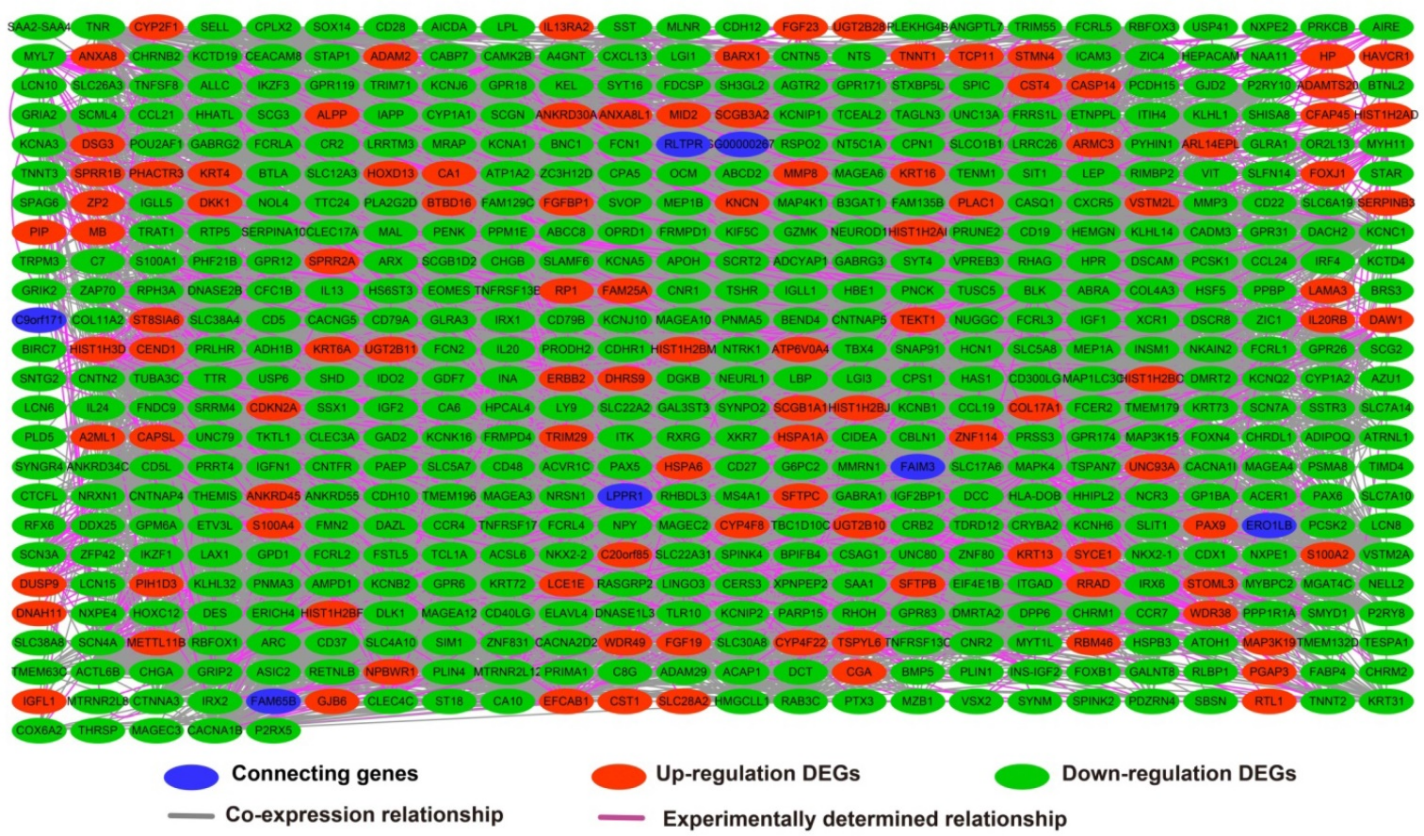
Protein-protein interaction regulatory network of DEGs between high- and low-LINP1 expression groups.

The edgeR software package of the R platform was used to screen 700 DEGs between low- and high-LINP1 expression groups, of which 560 were down-regulated and 140 were up-regulated DEGs (**Table S6**). GO term enrichment analysis of DEGs between different LINP1 expression groups suggested that these DEGs are involved in cell junction, the B cell receptor signaling pathway, cell-cell signaling, cell differentiation, phosphatidylinositol-4,5-bisphosphate 3-kinase activity, positive regulation of T cell proliferation, growth factor activity, and positive regulation of protein kinase B signaling (**Table S7**). The results of BiNGO suggested that these DEGs participate in cell-cell signaling, regulation of T cell activation, cell differentiation, regulation of T cell proliferation, regulation of B cell proliferation, cell junction, regulation of cell proliferation and regulation of cell communication (**Figure S2**). The results of KEGG analysis suggested that these DEGs are involved in cytokine-cytokine receptor interaction, the PPAR signaling pathway, adipocytokine signaling pathway, and metabolism of xenobiotics by cytochrome P450 (**Table S8**). The GGI (**Figure 7**) and PPI (**Figure 8**) analyses suggested that these DEGs have complex interaction regulatory networks.

**Figure 9:**
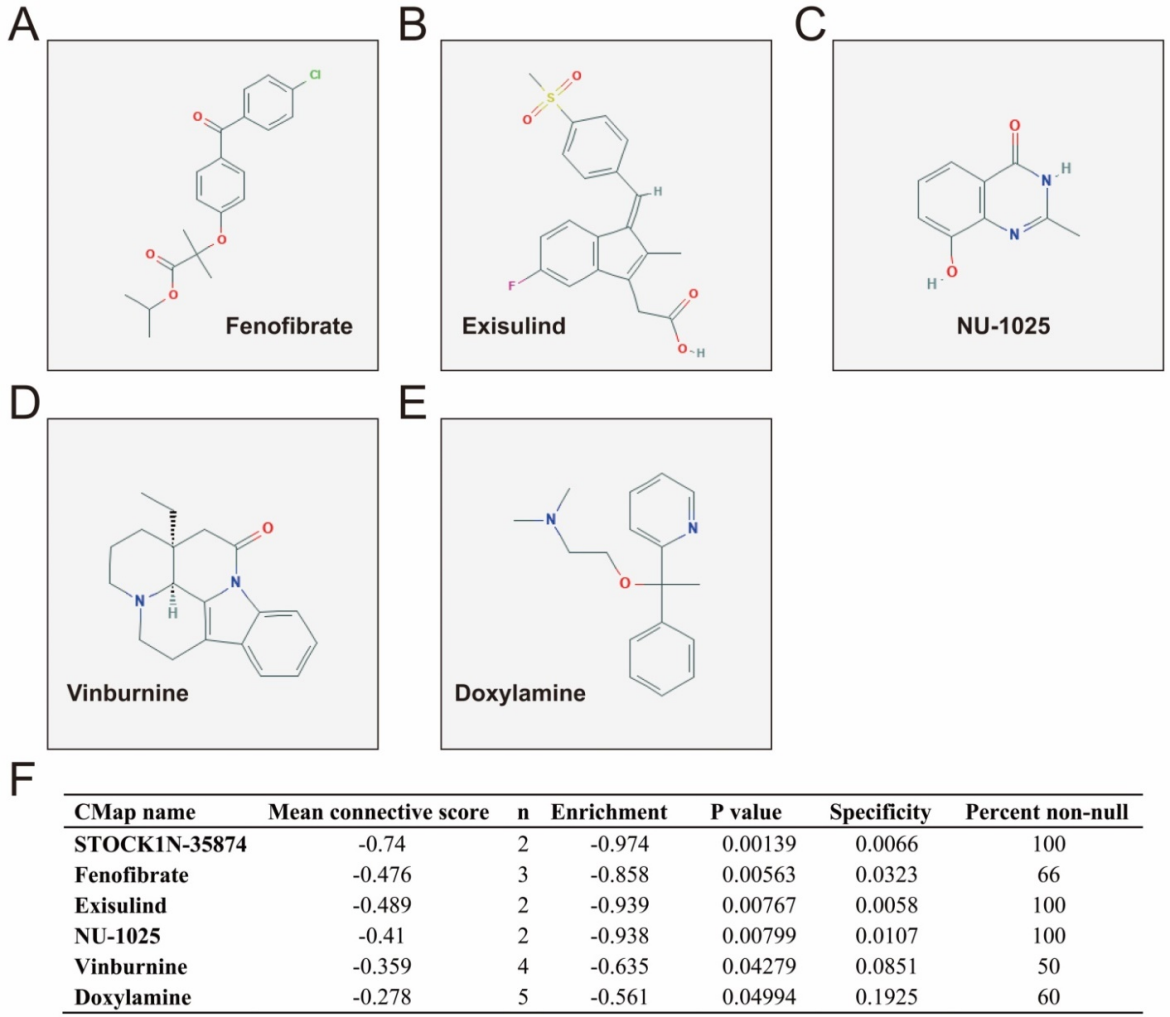
CMap analysis results and small molecule compound structure. Chemical structure of Fenofibrate (**A**), Exisulind (**B**), NU-1025 (**C**), Vinburnine (**D**), Doxylamine (**E**), and CMap analysis results (**F**). Notes: The structure of STOCK1N-35874 is not available on the PubChem database.

**Figure 10:**
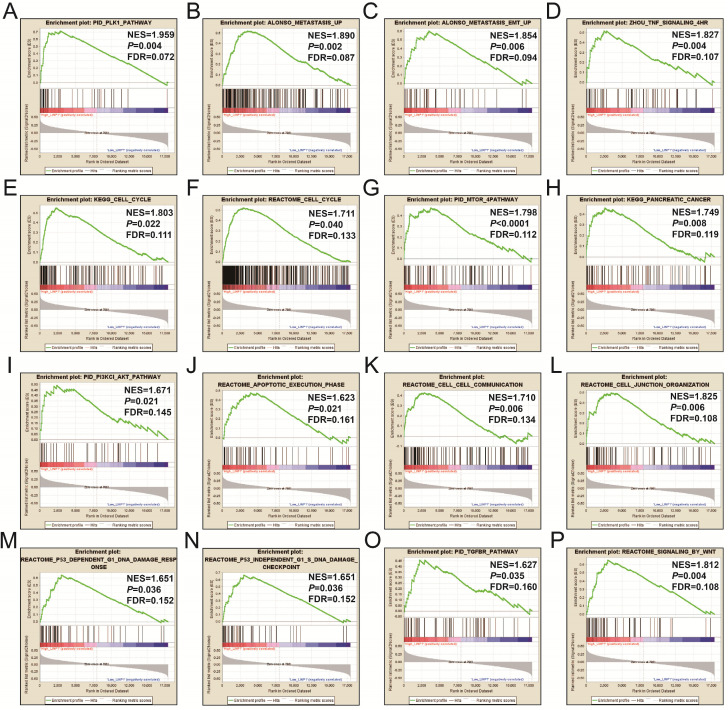
GSEA results of the high LINP1 expression group in early stage PDAC using the c5 reference gene set (**A-P**). (A), PID PLK1 PATHWAY; (B), ALONSO METASTASIS UP; (C), ALONSO METASTASIS EMT UP; (E),KEGG CELL CYCLE; (F),REACTOME CELL CYCLE; (G), PID MTOM 4 PATHWAY; (H), KEGG PANCREATIC CANCER; (I), PID PI3KCI AKT PATHWAY; (J), REACTOME APOPTOTIC EXECUTION PHASE; (K), REACROME CELL CELL COMMUNICATION; (L), REACROME CELL JUNCTION ORGANIZTION; (M), REACROME P53 DEPENDENT G1 DNA DAMAGE RESPONE; (N), REACROME P53 INDEPENDENT G1 S DNA DAMAGE CHECKPOINT; (O), PID TGFBR PATHWAY; (P), REACTOME SIGNALING BY WNT.

CMap analysis identified six small-molecule compounds that could be developed as potential LINP1 targeted drugs for the treatment of PDAC. The six small-molecule compounds were STOCK1N-35874, fenofibrate, exisulind, NU-1025, vinburnine, and doxylamine (**Figure 9**). To compensate for the deficiency of functional enrichment of DEGs, we explored the potential mechanism underlying the role of LINP1 in PDAC using a GSEA approach. When c5 (c5.all.v6.2.symbols.gmt) was used as a reference gene set, we were unable to obtain statistically significant results. Use of c2 (c2.all.v6.2.symbols.gmt) as a reference gene set indicated that LINP1 may be involved in the following pathways in PDAC: metastasis and epithelial-mesenchymal transition (EMT), cell cycle, pancreatic cancer, apoptosis execution phase, cell-cell communication, cell junction, p53 dependent G1 DNA damage response, p53 independent G1/S DNA damage checkpoint, class I phosphoinositide 3-kinase (PI3KCI)/Akt, polo-like Kinase 1 (PLK1), tumor necrosis factor (TNF), mammalian/mechanistic target of rapamycin (mTOR), transforming growth factor-beta receptor (TGFBR), and the Wnt signaling pathway (**Figure 10**, **Table S9**).

## Discussion

Liang et al. suggested that LINP1 acts as an oncogene in breast cancer. Breast cancer patients with high LINP1expression have shorter disease-free survival (DFS) and OS than those with low expression 9. Inhibition of LINP1 expression in breast cancer cell lines promotes apoptosis and induces cell cycle arrest. LINP1 is involved in distant metastasis in breast cancer by regulating EMT and the p53 pathway. LINP1 is expressed at higher levels in breast cancer patients with distant metastasis than in those without distant metastasis 9. The results of Liang et al. were confirmed by a study published by Liu et al., which indicated that breast cancer patients with high LINP1expression have a poor prognosis, and LINP1 is significantly overexpressed in breast cancer tissues 7. Zhang et al. used a clinically guided genetic screening approach in triple-negative breast cancer (TNBC) and found that LINP1 is not only highly expressed in TNBC tumor tissues, but also participates in the regulation of the non-homologous end joining (NHEJ) pathway through Ku80 and DNA-PKcs 28. LINP1 is also involved in the regulation of the p53 and EGFR signaling pathways, thereby affecting the sensitivity of breast cancer cell lines to radiotherapy 28. Similar studies were performed in cervical cancer. Wang et al. reported that LINP1 is involved in the NHEJ pathway by regulating Ku80 and DNA-PKcs in cervical cancer, and it modulates the radiation sensitivity of cervical cancer cells 29. LINP1 expression levels are notably increased in cervical cancer tumor tissues 29. Wu et al. observed that LINP1 acts as an oncogene in prostate cancer 8. The expression level of LINP1 is notably up-regulation in prostate cancer tumor tissues than in adjacent tumor tissues, and the OS time of high LINP1expression patients is notably shorter than these with low LINP1 expression 8. LINP1 expression is also related to T stage, lymph node metastasis, and distant metastasis in prostate cancer. Analysis of the underlying molecular mechanism showed that LINP1 regulates the malignant phenotype of prostate cancer cells through the p53 signaling pathway 8. De Silva et al. suggested that inhibiting the expression of LINP1 in TNBC cell lines blocks the interaction between insulin like growth factor binding protein 3 and non-POU domain containing octamer binding-splicing factor proline and glutamine rich, thereby affecting the DNA damage repair of cells. These results suggest that LINP1 affects the chemotherapy sensitivity of TNBC, and may be a therapeutic target for the treatment of TNBC 30. However, studies on the role of LINP1 in cancer have reported inconsistent results. Zhang et al. reported that LINP1 acts as a tumor suppressor gene in lung cancer, and silencing of LINP1 in lung cancer cell lines affects the migration, invasion, and stemness phenotypes of lung cancer cell lines by inhibiting EMT 31.

Regarding the six potential LINP1 targeted drugs for the treatment of PDAC, a literature review did not identify previous studies on the interaction between these drugs and LINP1. Most of these drugs, except doxylamine and vinburnine, were reported to have antitumor effects. In previous studies, CMAP analysis based on genome-wide expression profiling datasets identified STOCK1N‑35874 as a potential targeted therapy for colon adenocarcinoma and prostate carcinoma 32, 33. NU1025 is involved in regulating the sensitivity of human cervical cancer HeLa cells to the triazoloacridone compound c-1305 through the p53 pathway, thereby synergistically participating in tumor inhibition 34. Fenofibrate has anticancer effects in a variety of cancers 35, and it increases the sensitivity of tumors to chemotherapy and radiotherapy 36, 37. A literature review revealed that fenofibrate has anticancer effects in many malignancies, including pancreatic cancer 38, lung adenocarcinoma 39, hepatocellular carcinoma 40, melanoma 41, glioblastoma 42, 43, oral cancer 44, 45, prostate cancer 46, 47, breast cancer 48, neuroblastoma 49, and angiosarcomas 50. Fenofibrate also increases the sensitivity of esophageal carcinoma 51, 52 and head and neck squamous cell carcinoma 37 to radiotherapy, and that of breast cancer to chemotherapy 36. Exisulind is widely reported to have anticancer effects in a variety of malignancies, including non-small cell lung cancer 53, colon cancer 54-58, prostate cancer 59, breast cancer 60, head and neck squamous cell carcinoma 61, and pan-cancer 62. Low-dose celecoxib combined with exisulind can affect the tumorigenesis of prostate cancer by regulating pathways such as EGFR, Akt, androgen receptor, and cyclin D1. Exisulind was suggested as a potential drug for the prevention of prostate cancer 63.

Analysis of the potential functional mechanisms of LINP1 in PDAC showed that LINP1 is involved in the regulation of several cellular processes and pathways related to cell proliferation, cell cycle, and cell adhesion. Cell adhesion related biological processes and pathways are related to tumor metastasis and the malignant phenotype.

The present study has several limitations. First, because of the short survival time of the patients included in this study, the nomogram and time dependent ROC curve analysis were limited to 4 years. Second, because the study included a single cohort, the results need to be verified with multi-center studies and larger sample sizes. Third, the proposed underlying mechanisms need to be confirmed by performing *in vivo* and *in vitro* experiments. Nevertheless, the present study is the first to report the clinical application value and potential molecular mechanism of LINP1 in PDAC patients, as well as the screening of targeted drugs. The present study not only found the LINP1 can be used as a prognostic biomarker for PDAC, but also investigated the mechanism of LINP1 in PDAC by using TCGA genome-wide RNA-sequencing dataset. At the same time, CMAP was used to screen out the targeted drugs of LINP1 in PDAC. Once these findings are validated in multi-center dataset, and small molecule drugs are demonstrated in *in vivo* and *in vitro* experiments, our findings will provide important application values for prognosis prediction, postoperative surveillance management and treatment of PDAC.

## Conclusions

LINP1 may serve as a prognostic biomarker in early stage PDAC. Analysis of genome-wide co-expressed genes, DEGs screening, and GSEA approaches suggested that LINP1 is involved in the regulation of cell cycle, cell proliferation, and cell adhesion-related biological processes in PDAC. In addition, six small-molecule compounds including STOCK1N-35874, fenofibrate, exisulind, NU-1025, vinburnine, and doxylamine were identified as potential LINP1-targeted drugs for the treatment of PDAC. However, the present results need to be verified in future studies with larger sample sizes.

## Supplementary Material

Supplementary figures and tables.
