# Supplementary material for: Genome-wide RNA-sequencing dataset reveals the prognostic value and potential molecular mechanisms of lncRNA in non-homologous end joining pathway 1 in early stage Pancreatic Ductal Adenocarcinoma
Source: J Cancer. 2020 Jul 20;11(19):5556–67. doi: 10.7150/jca.39888 (PMC7477440; doi:10.7150/jca.39888)
Supplement: Supplementary file 1 — Supplementary figures and tables. [file jcav11p5556s1.zip › Supplementary material/Supplementary figures.pdf]

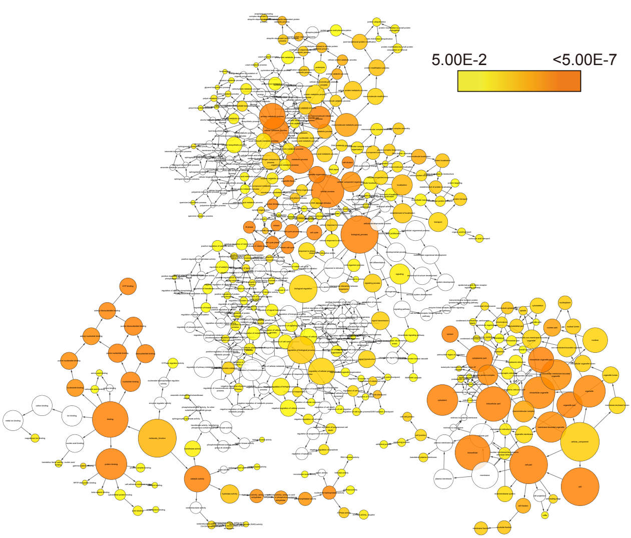

Figure S1. BiNGO enrichment results of LINP1 co-expressed genes.

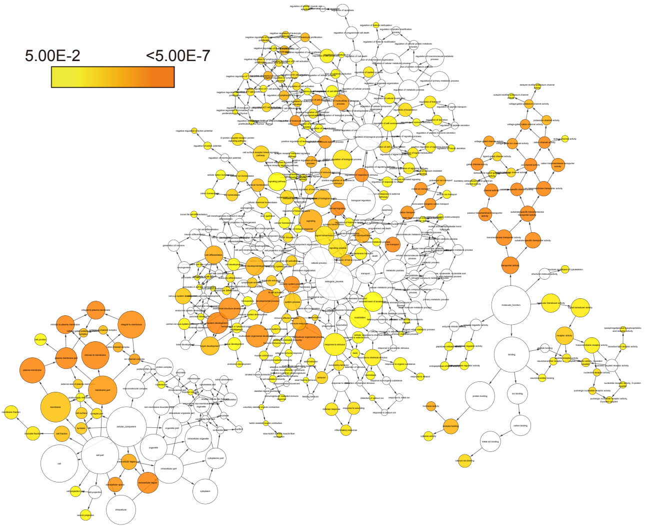

Figure S2. BiNGO enrichment results of DEGs between high- and low-LINP1 expression groups.
